# Case report: Fractional brain tumor burden magnetic resonance mapping to assess response to pulsed low-dose-rate radiotherapy in newly-diagnosed glioblastoma

**DOI:** 10.3389/fonc.2022.1066191

**Published:** 2022-12-06

**Authors:** Ryan F. Amidon, Fernando Santos-Pinheiro, Michael Straza, Melissa A. Prah, Wade M. Mueller, Max O. Krucoff, Jennifer M. Connelly, Christopher J. Kleefisch, Dylan J. Coss, Elizabeth J. Cochran, Joseph A. Bovi, Christopher J. Schultz, Kathleen M. Schmainda

**Affiliations:** ^1^ School of Medicine, Medical College of Wisconsin, Milwaukee, WI, United States; ^2^ Department of Neurology, Medical College of Wisconsin, Milwaukee, WI, United States; ^3^ Department of Radiation Oncology, Medical College of Wisconsin, Milwaukee, WI, United States; ^4^ Department of Biophysics, Medical College of Wisconsin, Milwaukee, WI, United States; ^5^ Department of Neurosurgery, Medical College of Wisconsin, Milwaukee, WI, United States; ^6^ Department of Biomedical Engineering, Marquette University and Medical College of Wisconsin, Milwaukee, WI, United States; ^7^ Department of Radiology, Medical College of Wisconsin, Milwaukee, WI, United States; ^8^ Department of Pathology, Medical College of Wisconsin, Milwaukee, WI, United States

**Keywords:** glioblastoma, pulsed low-dose-rate radiotherapy (pLDR), MRI, fractional tumor burden (FTB), relative cerebral blood volume (rCBV), pseudoprogression, treatment effect, tumor progression

## Abstract

**Background:**

Pulsed low-dose-rate radiotherapy (pLDR) is a commonly used reirradiation technique for recurrent glioma, but its upfront use with temozolomide (TMZ) following primary resection of glioblastoma is currently under investigation. Because standard magnetic resonance imaging (MRI) has limitations in differentiating treatment effect from tumor progression in such applications, perfusion-weighted MRI (PWI) can be used to create fractional tumor burden (FTB) maps to spatially distinguish active tumor from treatment-related effect.

**Methods:**

We performed PWI prior to re-resection in four patients with glioblastoma who had undergone upfront pLDR concurrent with TMZ who had radiographic suspicion for tumor progression at a median of 3 months (0-5 months or 0-143 days) post-pLDR. The pathologic diagnosis was compared to retrospectively-generated FTB maps.

**Results:**

The median patient age was 55.5 years (50-60 years). All were male with IDH-wild type (n=4) and O^6^-methylguanine-DNA methyltransferase (MGMT) hypermethylated (n=1) molecular markers. Pathologic diagnosis revealed treatment effect (n=2), a mixture of viable tumor and treatment effect (n=1), or viable tumor (n=1). In 3 of 4 cases, FTB maps were indicative of lesion volumes being comprised predominantly of treatment effect with enhancing tumor volumes comprised of a median of 6.8% vascular tumor (6.4-16.4%).

**Conclusion:**

This case series provides insight into the radiographic response to upfront pLDR and TMZ and the role for FTB mapping to distinguish tumor progression from treatment effect prior to redo-surgery and within 20 weeks post-radiation.

## Introduction

Glioblastoma is a primary central nervous system glioma designated as World Health Organization (WHO) grade 4 with wildtype isocitrate dehydrogenase (IDH-wt) (1). Patients with glioblastoma have a median overall survival of 12-15 months following diagnosis, with a five-year survival between 3 and 5.5% (2–5). The standard of care for glioblastoma includes surgical resection, chemoradiotherapy with temozolomide (TMZ) followed by adjuvant TMZ and tumor-treating fields (TTF) (5–9). Response to treatment is routinely assessed by magnetic resonance imaging (MRI). Pulsed low-dose-rate radiotherapy (pLDR) is a commonly used reirradiation technique for recurrent high-grade gliomas, but its upfront use with concurrent TMZ is currently under investigation (10–13). While other salvage therapies for recurrent high-grade glioma exist, including stereotactic radiosurgery, conformal external beam radiation, and brachytherapy, pLDR delivers radiation in subfractions at specific time intervals, taking advantage of the hyper-radiosensitivity of proliferating tumor cells to low doses of radiation, as well as the reduced toxicity to normal brain tissue (12, 14). Currently, there is limited data on the radiographic response to pLDR.

Distinguishing between treatment effect and tumor progression is challenging on standard imaging, with a definitive diagnosis only possible with pathologic confirmation. Radiotherapy may induce an inflammatory intraparenchymal response with subsequent necrosis and/or edema that is indistinguishable from, or intermingled with, tumor progression on MRI (15). Specifically, postcontrast MRI highlights blood-brain barrier disruption, which can be observed with both non-tumor and viable tumor tissue (15). Therefore, a new contrast-enhancing lesion arising within the radiation field of a treated glioblastoma can neither confirm nor refute progression of disease (16). A recent meta-analysis identified treatment effect in 36% of high-grade glioma cases (17). More recently, certain tumor and treatment factors have correlated with increased observation of treatment effect on MRI, including O^6^-methylguanine-DNA methyltransferase (MGMT) promoter methylation, radiation dose, dose per fraction, treatment duration, irradiated brain volume, and concurrent use of TMZ (18–22).

Fractional tumor burden (FTB) mapping is a novel radiographic biomarker that spatially distinguishes viable high-grade tumor from treatment effect within postcontrast T1-weighted enhancement on MRI (23). These maps are generated from dynamic susceptibility contrast (DSC) MR perfusion, which allows for the visualization of regional cerebral blood volume (RCBV) to identify neovascularization. FTB maps use tissue-validated standardized regional cerebral blood volume (sRCBV) thresholds (23, 24) and assign primary colors to different perfusion patterns: treatment effect (blue; sRCBV < 1.0), tumor/treatment effect admixture (yellow; 1.0 > sRCBV < 1.6), and viable tumor (red; sRCBV > 1.6). Standardized thresholds are advantageous in that they require minimal user input compared to thresholds normalized based on user-defined reference tissues (25). It was demonstrated that regions of high-grade vascular tumor (yellow + red) could be distinguished from regions of treatment effect (blue) with a sensitivity/specificity of 79.4%/90% and an accuracy of 85% (23). Hoxworth et al. prospectively validated FTB maps with tissue, observing an 85% accuracy of identifying voxels with at least 50% viable tumor (25). Of note, Iv et al. determined that either the red or blue regions were best at distinguishing tumor from treatment effect, using a different sRCBV threshold of 1.75 (26).

In this case series, we present four patients with glioblastoma treated with upfront pLDR with concurrent TMZ following maximal safe resection. Treatment response was assessed by 3 Tesla (3T) MRI with and without contrast, FTB maps, and radiographic findings were confirmed by pathology.

## Case descriptions

### Case 1

A 50-year-old male presented with a progressive cognitive decline, expressive aphasia, and personality changes. Brain MRI revealed a non-enhancing small region of increased T2-weighted signal in the right frontal lobe; a follow-up MRI 25 weeks later revealed an avidly enhancing mass exhibiting significant mass effect. He was eventually submitted for craniotomy and gross-total resection of the enhancing lesion. Integrated diagnosis was consistent with glioblastoma, WHO grade 4, IDH-wt, and MGMT hypermethylated. At four-weeks post-surgery, there was improvement in cognitive performance although personality changes persisted. He was treated with pLDR as part of a prospective phase II study (NCT04747145) to a total dose of 60 Gray (Gy) in 30 fractions with concurrent TMZ, which he tolerated with anticipated side effects. This was followed by adjuvant TMZ and TTF.

**Figure 1:**
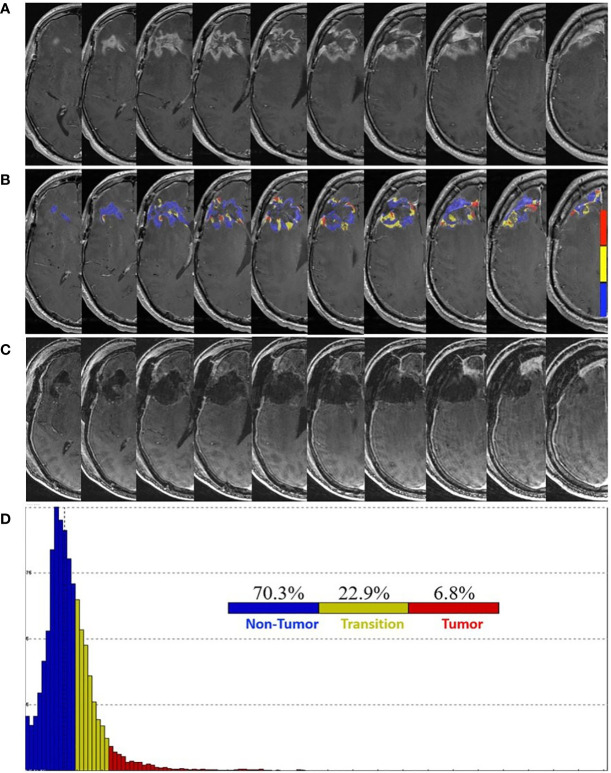
Pre- and post-surgery MRI. **(A)** Postcontrast T1-weighted MRI obtained 20 weeks following the completion of pLDR and two weeks before redo-surgery. **(B)** Corresponding maps of FTB superimposed on the postcontrast T1-weighted MRI images. The FTB maps show areas of active tumor (red), treatment effect (blue), and transitional zone (yellow). **(C)** Immediate post-operative postcontrast T1-weighted MRI demonstrating extent of resection. **(D)** Distribution of T1-weighted enhancement volume based on vascularization as measured by FTB maps.

The patient completed a total of four cycles of TMZ and TTF before MRI of the brain, obtained at 20 weeks post-pLDR, which raised concerns for tumor progression. Increased nodular enhancement around the resection cavity (**Figure 1A**) was observed in the setting of the patient experiencing neurocognitive decline. FTB maps revealed predominantly treatment effect with 70.3% non-tumor, 22.9% tumor admixture, and 6.8% tumor tissue (**Figures 1B, D**).

At 23 weeks post-pLDR, the patient was submitted for redo-craniotomy in the absence of FTB-guidance. Near-total resection of the enhancing lesion was later confirmed by postoperative MRI (**Figure 1C**). Microscopy of the five tissue specimens revealed a combination of necrosis, hyalinized and necrotic blood vessels, chronic inflammation, foamy macrophages, mineralization, and reactive gliosis, consistent with treatment effect. Therefore, the patient resumed adjuvant treatment with TMZ and TTF. Of note, compliance with TTF averaged 90% of “ON” time throughout the treatment period. At four weeks post-redo surgery, the patient remained clinically stable.

### Case 2

A 56-year-old male presented with a history of seizure and expressive aphasia. Brain MRI revealed a left parietal mass with surrounding vasogenic edema and mass effect. Craniotomy and gross-total resection were performed, and integrated diagnosis was consistent with glioblastoma WHO grade 4, IDH-wt, and MGMT unmethylated. The patient was treated with pLDR as a part of a prospective phase II study (NCT04747145) to a total dose of 60 Gy in 30 fractions with concurrent TMZ followed by adjuvant TMZ and TTF.

**Figure 2:**
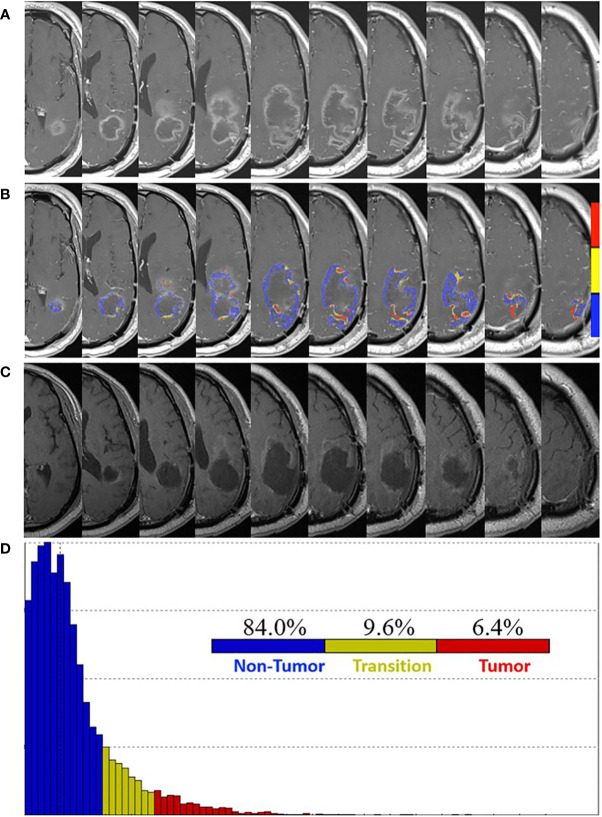
Pre- and post-surgery MRI. **(A)** Postcontrast T1-weighted MRI obtained five weeks post-pLDR and two weeks before redo-surgery. **(B)** Corresponding maps of FTB superimposed on the postcontrast T1-weighted MRI images. The FTB maps show areas of active tumor (red), treatment effect (blue), and transitional zone (yellow). **(C)** Post-surgery (four weeks) postcontrast T1-weighted MRI demonstrating extent of resection. **(D)** Distribution of T1-weighted enhancement volume based on vascularization as measured by FTB maps.

At one week post-pLDR, there was clinical decline with MRI demonstrating significant vasogenic edema for which a bevacizumab infusion was provided every two weeks at 10 mg/kg. It was discontinued after two infusions due to a poor response and continued clinical decline. At five weeks post-pLDR, MRI demonstrated an increase in the left parietal peripherally enhancing lesion size (**Figure 2A**). FTB maps revealed predominantly treatment effect within the contrast enhancement surrounding the resection cavity with 84.0% non-tumor, 9.6% tumor admixture, and 6.4% tumor (**Figures 2B, D**).

At eight weeks post-pLDR, redo-craniotomy was performed in the absence of FTB-guidance. Reduced vasogenic edema and regions of enhancement around the resection cavity were later confirmed by MRI performed four weeks post-surgery (**Figure 2C**). Three tissue specimens were evaluated, two of which showed necrosis and hyalinized vessels, consistent with treatment effect, while the last sample revealed hypercellular high-grade glioma. The patient continued adjuvant treatment with TMZ and TTF. At nine weeks post-redo surgery, the patient’s neurological symptoms included fatigue, right hemiparesis, and global aphasia. At 10 weeks post-redo surgery, he was restarted on bevacizumab at 10 mg/kg every 2 weeks, completing two infusions. Ultimately, he experienced a traumatic fall complicated by intraparenchymal hemorrhage and was transitioned to hospice.

### Case 3

A 55-year-old male presented with a seizure, visual impairment and personality changes. Brain MRI revealed an enhancing left occipital mass surrounded by diffuse FLAIR hyperintensity signal. Craniotomy and gross-total resection were performed and integrated diagnosis was consistent with glioblastoma WHO grade 4, IDH-wt, and MGMT unmethylated. Following surgery, his symptoms included right hemiparesis, global aphasia, and a right homonymous hemianopsia. The patient was enrolled in a prospective phase II study (NCT04747145) to be treated with pLDR to a total dose of 60 Gy in 30 fractions with concurrent TMZ followed by adjuvant TMZ and TTF.

**Figure 3:**
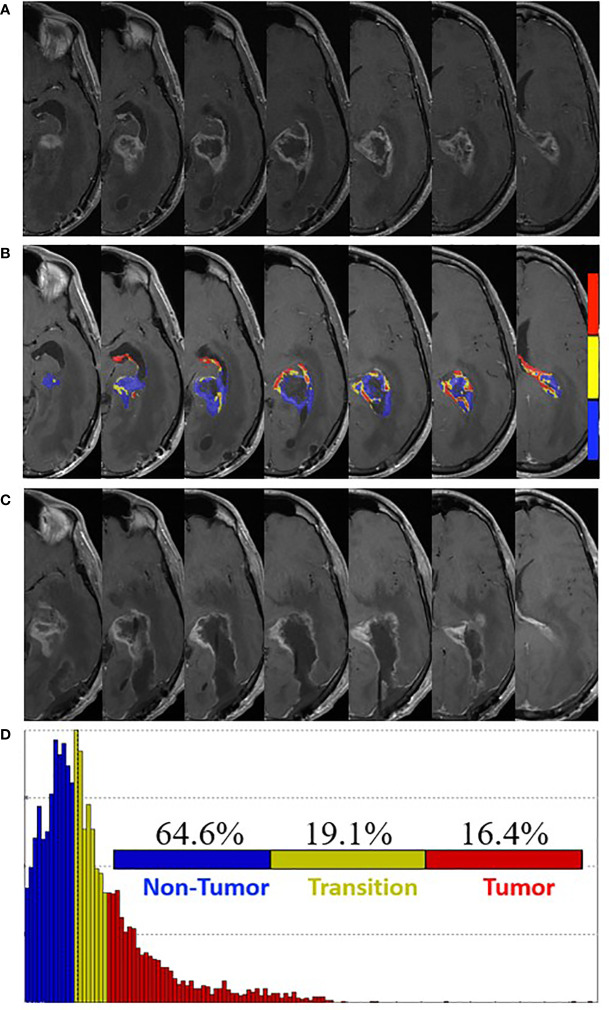
Pre- and post-surgery MRI. **(A)** Postcontrast T1-weighted MRI obtained four weeks post-pLDR treatment and one week before redo-surgery. **(B)** Corresponding maps of FTB superimposed on the postcontrast T1-weighted MRI images. The FTB maps show areas of active tumor (red), treatment effect (blue), and transitional zone (yellow). **(C)** Post-surgery (one week) postcontrast T1-weighted MRI demonstrating extent of resection. **(D)** Distribution of T1-weighted enhancement volume based on vascularization as measured by FTB maps.

Near completion of pLDR (fraction 24) and 8 weeks post-surgery, he was admitted for worsening symptoms of gait dysfunction, nausea and vomiting, and dysphagia. MRI revealed increased enhancement around the resection cavity with increased mass effect and left ventricular trapping (**Figure 3A**). FTB maps revealed predominantly treatment effect within the contrast enhancement surrounding the resection cavity with 64.6% non-tumor, 19.1% tumor admixture, and 16.4% tumor (**Figures 3B, D**). Radiation was withheld and redo-craniotomy was performed in the absence of FTB-guidance. Five tissue specimens revealed necrotic tissue and sparse reactive brain tissue, consistent with treatment effect. Neurologically, the patient improved in memory and speech with residual comprehensive aphasia and right visual field defect. Bevacizumab was subsequently started at 10 mg/kg every 2 weeks.

The patient continued adjuvant treatment with bevacizumab, TMZ, and TTF. Of note, compliance with TTF averaged 75-90% of “ON” time throughout the treatment period. At one week post-surgery, brain MRI demonstrated retraction of the resection cavity and decreased peripheral enhancement along the surgical margins (**Figure 3C**). At 29 weeks post-redo surgery, the patient was transitioned to hospice, experienced respiratory failure caused by aspiration from worsening dysphagia and expired.

### Case 4

A 60-year-old male presented with progressive left hemiparesis and memory decline. Brain MRI revealed an enhancing mass within the right frontal lobe that was resected. Integrated diagnosis was consistent with glioblastoma WHO grade 4, IDH-wt, and MGMT unmethylated. There was significant postoperative improvement in symptoms. The patient was treated with pLDR as a part of a prospective phase II study (NCT04747145) to a total dose of 60 Gy in 30 fractions with concurrent TMZ followed by adjuvant TMZ. Adjuvant TTF were declined.

**Figure 4:**
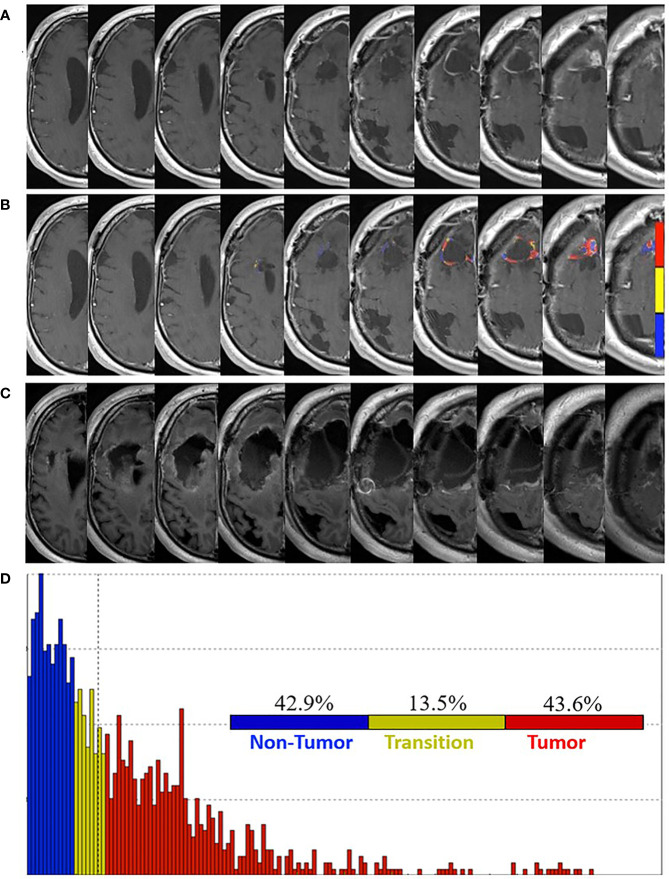
Pre- and post-surgery MRI. **(A)** Postcontrast T1-weighted MRI obtained 20 weeks post-pLDR and 10 weeks before redo-surgery. **(B)** Corresponding maps of FTB superimposed on the postcontrast T1-weighted MRI images. The FTB maps show areas of active tumor (red), treatment effect (blue), and transitional zone (yellow). **(C)** Immediate post-operative postcontrast T1-weighted MRI with FLAIR demonstrating extent of resection, which encompasses regions of tumor identified in the FTB maps. **(D)** Distribution of T1-weighted enhancement volume based on vascularization as measured by FTB maps.

Follow-up brain MRI studies demonstrated progressively increasing enhancement around the resection cavity. At 13 weeks post-pLDR, FTB maps revealed 47.9% non-tumor, 19.2% tumor admixture, and 32.9% tumor within the contrast-enhancing tissue. Seven weeks later, brain MRI demonstrated increased enhancement, particularly at the superomedial resection cavity margin (**Figure 4A**). FTB maps were again obtained with increasing proportions of vascular tumor tissue suggesting progression: 42.9% non-tumor, 13.5% tumor admixture, and 43.6% tumor (**Figures 4B, D**).

At 30 weeks post-pLDR, redo-craniotomy was performed in the absence of FTB-guidance. Significant debulking was later confirmed by MRI (**Figure 4C**). Two tissue specimens revealed moderately cellular atypical glial proliferation, supportive of active tumor. At three weeks post-redo surgery, the patient progressed with gait dysfunction, dysphagia, expressive aphasia, and left facial droop.

## Discussion

This case series describes examples of how FTB can improve radiologic accuracy when used as a biomarker to assess treated glioblastoma. In one study, participating physicians determined that they would change treatment in 93% of cases where the tumor fraction was predominant and not change treatment in 75% of cases where treatment effect was predominant, based on their interpretation of FTB maps (26). The authors suggested the potential use of FTB for providing guidance in deciding which patients need an operation (26). In all four of the cases presented here, there were concerns about tumor progression based on standard 3T MRI. Pathologic examination revealed treatment effect in two cases and viable tumor in the other two cases. FTB maps were indicative of lesion volumes being comprised of predominantly treatment effect in three cases and predominantly viable tumor in one case. From the three FTB maps in the former category, the median fraction of the enhancing tumor volume comprised of vascular tumor was 6.8% (range 6.4-16.4%).

Radiologic assessment of treated glioblastoma remains a challenge as an indistinguishable MRI pattern between treatment effect and viable tumor may develop in about 36% of these cases (17). Often, with no other options, biopsy or surgery is pursued for diagnostic confirmation, posing potentially unnecessary risks. This common scenario highlights the need for more accurate radiologic biomarkers that not only guide treatment but also prevent unnecessary surgical intervention. This is of particular importance with heterogenous tumors such as glioblastoma that are more prone to sampling error. FTB maps provide meaningful information regarding the spatial distribution of tumor and treatment effect within enhancing lesions and may be able to fill that gap.

There are several limitations to this study. FTB maps were generated retrospectively and therefore not used for surgical guidance or tissue confirmation in none of the four cases. It also does not provide information about the clinical course of the disease. Monitoring FTB over time may provide additional information to evaluate this, as demonstrated in Case 4. We also assume that the pathology reports are always accurate, when sampling bias may occur. For instance, it is unknown whether an active tumor is in the process of dying or may die in the near future, especially if it is sampled too soon following radiation. In parallel, bevacizumab as a treatment for symptomatic radiation necrosis in Cases 2 and 3 may have confounded their respective radiologic responses to pLDR, as it may reduce the area of enhancing necrosis by decreasing vascular permeability and inflammation (27). Finally, the small sample size from our single institution limits generalizability.

This case series provides insight into response to upfront pLDR concurrent with TMZ following resection of newly diagnosed glioblastoma. It also highlights the capacity of FTB maps to accurately distinguish tumor progression from treatment effect. Therefore, full validation of FTB mapping as a biomarker should be pursued as a prospective study with larger sample size.

## Data availability statement

The original contributions presented in the study are included in the article/supplementary material. Further inquiries can be directed to the corresponding author.

## Ethics statement

The studies involving human participants were reviewed and approved by Medical College of Wisconsin Institutional Review Board. The patients/participants provided their written informed consent to participate in this study. Written informed consent was obtained from the individual(s) for the publication of any potentially identifiable images or data included in this article.

## Author contributions

FS-P, MS, WM, and MK treated the patients. DC and EC analyzed tissue samples and generated pathology reports for the patients. RA wrote the first draft of the manuscript and generated figures. MP and KS performed image processing and generated the FTB maps. MP, MS, FS, WM, MK, CK, DC, EC, JB, CS, and KS edited the paper. All authors contributed to the article and approved the submitted version.
